# The role of registered nurses in primary care and public health collaboration: A scoping review

**DOI:** 10.1002/nop2.496

**Published:** 2020-04-14

**Authors:** Monica Swanson, Sabrina T. Wong, Ruth Martin‐Misener, Annette J. Browne

**Affiliations:** ^1^ School of Nursing University of British Columbia Vancouver BC Canada; ^2^ School of Nursing and Centre for Health Services and Policy Research University of British Columbia Vancouver BC Canada; ^3^ School of Nursing Dalhousie University Halifax NS Canada; ^4^ Present address: North Island College Courtenay BC Canada

**Keywords:** collaboration, nurses, nursing, primary health care, public health

## Abstract

**Aim:**

The purpose of this work was to examine the roles of Registered Nurses (RNs) in primary care (PC) and public health (PH) collaboration. Additionally, we aimed to explore whether the current scope of practice for RNs is adequate to support their roles in PC/PH collaboration.

**Design:**

A scoping review of current literature relating to the RN’s role in PC/PH collaboration was conducted using the PRISMA 2009 checklist.

**Methods:**

The review used key terms: primary care, public health, collaboration, nursing and nurse role across six electronic databases; 23 articles that were included in the final review were published over a 7‐year span.

**Results:**

Four key RN roles relating to PC/PH collaboration were identified: relationship builder, outreach professional, programme facilitator and care coordinator. RNs supported transitions in chronic disease, communicable disease care and maternity care at various healthcare system levels including systemic, organizational, intrapersonal and interpersonal levels.

## 1 INTRODUCTION

Worldwide, attention is being given to the transformation of healthcare systems from an illness focus to one that is person‐centred and health‐promotive (World Health Organization (WHO), 2016), with calls for improving access to high‐quality collaborative care (Farmanova et al., 2016), especially in community‐based primary health care (Organization for Economic Co‐operation and Development (OECD), 2017; WHO, 2016). Healthcare systems with poor collaboration are inefficient, expensive and not well equipped to handle the potential “tsunami wave effect” of chronic diseases such as heart disease, diabetes, cancer and mental illness (Millar, Bruce, Cheng, Masse, & McKeown, 2013) in addition to episodic crises such as avian flu or fentanyl overdose epidemics.

## 2 BACKGROUND

There are increasing calls for the improvement of collaborative care, which would promote optimal patient care. One way to improve the effectiveness of the healthcare system is collaboration between primary care (PC) and public health (PH) (Institute of Medicine, 2012; Strumpf et al., 2012; Valaitis et al., 2013). The public health system, generally, is legally driven to administer communicable disease management, environmental monitoring and health promotion. Primary care is responsible for being the “first door” to the healthcare system, providing generalized health care throughout the lifespan from infancy to older adulthood. Evidence suggests that collaboration between PC and PH systems can be especially beneficial in circumstances where care delivery and management is complex (Valaitis et al., 2013). Communicable disease management such as influenza, chronic disease care such as diabetes and asthma, complex maternal/child health care and care delivery to vulnerable populations experiencing health and social inequities are examples of areas of care that would benefit from collaboration between PC and PH. However, health care can be fragmented and discontinuous, making PH/PC collaboration difficult (Hutchison, Levesque, Strumpf, & Coyle, 2011). Collaboration for the purposes of this paper means healthcare professionals assuming complementary roles and working together towards a common goal enhanced patient care (O’Daniel & Rosentein, 2008). Workforce analysis of who would do this collaborative work falls on physicians and nurses as the largest health professionals in the two systems. Based on their size in numbers, Registered Nurses (RNs), Licensed Practical Nurses (LPNs) and Nurse Practitioners (NPs) could play an important role in strengthening collaboration between PC and PH (Valaitis et al., 2013).

Nurses constitute the largest workforce in PH and increasingly in PC (Ammi, Ambrose, Hogg, & Wong, 2017; Wong et al., 2015). Based on their scope of practice, which includes health promotion, collaboration and chronic disease care (Australian Primary Health Care Nurses Association, 2017; British Columbia College of Nursing Professionals, 2018), nurses should be considered in primary healthcare system renewal (Fraher, Spetz, & Naylor, 2015; Smolowitz et al., 2015).

There is a clear need to clarify and optimize the RN’s role in PC/PH collaboration (Bauer & Bodenheimer, 2017; Fraher et al., 2015; Halcomb, Stephens, Bryce, Foley, & Ashley, 2016; Martin‐Misener & Bryant‐Lukosius, 2014), using their skills in health promotion and disease prevention and collaboration. Yet, the lack of RN role clarification and competency standards in this area (Halcomb et al., 2016; Registered Nurses Association of Ontario, 2012) and structural challenges such as restrictive funding models that inhibit team‐based care (Bauer & Bodenheimer, 2017; Hutchinson et al., 2011) impedes the uptake of the RN role in these areas (Martin‐Misener & Bryant‐Lukosius, 2014).

The purpose of this work was to examine the roles of RN in PC/PH collaboration in relation to their scope of practice. Specifically, we answer the following questions: (a) What are the roles of RNs in PC/PH collaboration; and (b) is the current scope of practice for RNs adequate to support their roles and activities in PC/PH collaboration? This research focused specifically on RN degree‐prepared nurses; other nursing designations such as NPs and LPNs were not included in this review due to RNs’ size in numbers and potential impact on the healthcare system and the differing scopes of practice of NPs and LPNs.

## 3 DESIGN

A scoping review of the RN’s role in PC/PH collaboration was conducted. One of the goals of using a scoping review is to further enhance understanding, applicability and refinement of research questions that are broad and complex or have not been studied, for example RNs’ role in collaboration between PC and PH in this research (Arksey & O’Malley, 2005; Colquhoun et al., 2014). Using this methodology illuminates the RN’s role in PC/PH collaboration and may assist in the development of future research questions. The PRISMA checklist (see File S1) provided support for reporting findings.

## 4 METHODS

Drawing on the work of Arksey and O’Malley (2005), Levac, Colquhoun, and O’Brien (2010) and Valaitis et al. (2012), we followed six steps in conducting our research: identifying the research questions; searching for relevant studies; selecting studies; charting the data and collating; summarizing; and reporting the results.

### 4.1 Search strategy and study selection

Our database search consisted of: PubMed, CINAHL; Cochrane; PsycInfo; Sociological Abstracts; Web of Science; and Dissertation International. We also scanned reference lists of included articles and conducted Web searches of government, healthcare associations and research networks for key documents and information. Finally, a general Internet search using key terms was used to capture grey literature and/or other information not gained from formal databases. Key terms used in the search included the following: primary care, public health, collaboration, public health nurse, community health nurse, nurse, patient care teams and nurse's roles. A health science librarian at the University of British Columbia supported this scoping review (Appendix S1).

**Figure 1:**
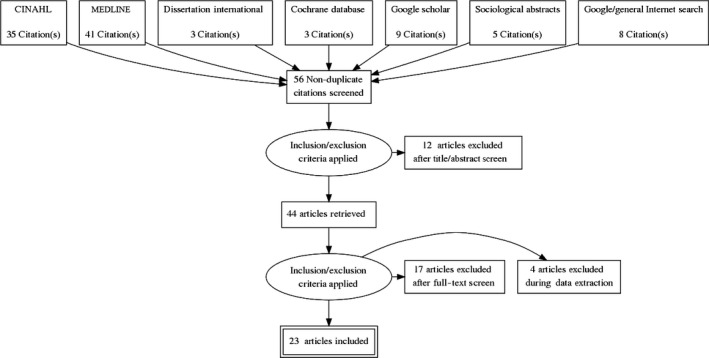
PRISMA Diagram

Papers addressed at least one of the following: structures and processes supporting RN’s role in PC/PH collaboration or patient population indicators and outcomes of RN collaboration between PC and PH. Papers were excluded if they addressed PH or PC alone; contained no evidence of collaboration; did not describe the RN’s role in collaboration; or were not published in English (Figure 1). Articles published between January 2009– January 2016 were screened for applicability. These dates were chosen to build on the Canadian scoping review completed by Martin‐Misener et al. (2012) and to capture additional international literature around PC/PH RN implementation. Literature published from 2016–present (Pratt et al., 2018; Valaitis, Meagher‐Stewart, Martin‐Misener, Wong, & MacDonald, 2018; Wong et al., 2017) provides more information on systemic and organizational considerations in PC/PH collaboration; however, they do not specifically address the nurse role.

Papers were from the USA, Canada, Western Europe, Australia, the UK and New Zealand. Commentaries were included if they brought context to the research question. In this scoping review, limited grey literature was retrieved and did not inform this study.

### 4.2 Analysis

Data were extracted using the concept of “charting the data” as suggested in Arksey and O’Malley (2005). This process is similar to data extraction in a systematic review but involves taking a comprehensive approach to the material and compiling it in a manner that contextualizes the outcomes; this makes it more suitable to readers who will use the findings to inform practice and policy decisions. Data were coded into the following categories: type and/or purpose of collaboration; participants involved in the collaboration; RN’s role in the collaboration; geographic context/situation (urban/rural); health focus such as mental health, maternal or communicable disease; motivators for collaboration; characteristics and attributes of collaboration; and results and indicators of success. We used an interpretive description approach (Thorne, 2016) to analyse the coded data and formulate themes.

Data pertaining to the RN’s role in PC/PH collaboration were also analysed using intrapersonal, interpersonal, organizational and systemic levels identified in the Ecological Framework for Building Successful Collaboration between Primary Care and Public Health (Appendix S2; Valaitis et al., 2013). This framework identifies supporting factors at each level that enhance collaboration between PH and PC sectors. It also provided guidance in the data analysis phase of by allowing us to understand the RN’s role in the context of collaboration and from a lens that identifies collaboration beyond the interpersonal and intrapersonal levels. To ensure credibility and study rigour, data coding and analysis were reviewed by the study team throughout. To examine whether reported RN roles in PC/PH collaboration were in their general scope of practice, we examined these roles against the example of British Columbia's RN scope of practice.

## 5 ETHICS

In conducting this scoping review, no research ethics committee approval or patient consent was needed and therefore not obtained. No funding was obtained through this research by the authors or any conflict of interest identified.

## 6 RESULTS

The combined search strategy yielded a total of 56 papers, and of these, 23 papers met the inclusion criteria (Figure 1). Twelve papers were removed as there was no clear collaboration between PC and PH. The remaining 44 papers were read in detail, and 21 papers were excluded for no identification of nurse role. Most papers were published from 2012–2014 (see Table 1).

**TABLE 1:** Papers included in the scoping review

Author/Year	Title	Purpose	Main Findings	Participants	Methods	Country	Rural, urban, both/none
Bodenheimer et al. (2009)	Confronting the Growing Burden of Chronic Disease	Chronic disease prevention strategies/ workforce planning	Using nurses in PC for chronic disease management	Primary care and public health	Commentary	USA	None
Clancy et al. (2013)	PHN and interprofessional collaboration in Norwegian municipality	Collaboration and PHNs in different sized communities	Collaborative roles between PH and PC	PHNs, doctors, midwives	Survey	Norway	Both
Elliot et al. (2014)	Health care system collaboration to address chronic diseases: a nationwide snapshot from state public health practitioners	Collaboration of public health and chronic disease management	RNs providing toolkits and training to PC around chronic disease	Public health practitioners	National Survey	USA	Both
Ferrer et al. (2013)	Advanced primary care in San Antonio: linking practice and community strategies…	Community of solution concept target vulnerable population	RNs supporting care transition and case management	Public health Primary care Community workers	Commentary	USA	Urban
Green et al. (2013)	Improving collaboration. Between PH and family health teams in Ontario	Identification of areas of overlap of PC teams and PH	Role overlap between PC and PH	Family health team Public health	Mixed‐methods survey/interviews	Canada (Ontario)	Urban
Kates et al. (2012)	Framework for advancing improvement in primary care	PC Improvement	Team‐based care—increase health promotion work	None	Commentary	Canada (Ontario)	None
Kelly et al. (2015)	PHN experiences of their role in PC	PHN views and experiences as part of a PCT	Role clarification and loss of health promotion in PC	PHNs	IPA qualitative	IRE	Both
Kempe et al. (2014)	Effectiveness of PC PH collaboration in influenza delivery	PHN views and experiences as part of a PCT	Improving influenza vaccination rates through PC/PH RN collaboration	PC sites and PH	RCT with qualitative approach	USA	Urban
Korhonen et al. (2014)	Primary care‐based, targeted screening program to promote sustained weight management	Weight interventions	Home‐based PH screening and PC collaboration	Patients with PHN intervention	Survey/questionnaires	Finland	Rural
Monsen et al. (2015)	Translation of obesity practice guidelines: interprofessional perspectives regarding the impact of public health nurse system‐level intervention	Guideline supports PH to PC	RN role in system‐level obesity prevention reduced siloed care	Admin and clinicians LPN, RN, PHN, NP, MD, dietician, PT/OT	Qualitative	USA	None
Psaila, Kruske, et al. (2014)	Qualitative study of innovations for transition care	Innovations to improve transition of care for maternity patients	Nurse liaison role	Child family health nurse, midwife, GP, support workers, social workers	Mixed‐methods phase 3 interviews	AUS	None
Psaila, Kruske, et al. (2014)	Smoothing out the transition of care between maternity and child and family health services: perspectives of child and family health nurses and midwives	Transition of care primary care midwifery to child family health nurses	Importance of transition of care—liaison role	Midwife/ child family health nurses	Mixed‐methods phase 2 ‐survey	AUS	None
Psaila, Kruske, et al. (2014)	Discontinuities between maternity and child and family health services: health professional's perceptions	Concept of continuity of maternity services	Child health nurse role in collaboration with PC improved patient experience	Midwife, child family health nurses, GP, practice nurses	Interviews	AUS	None
Wynn and Moore (2012)	Integration of primary health care and public health during a public health emergency	HINI	PH RNs were supplemental providers with PC in outbreak management	PC staff PH staff	Commentary	Canada (Ontario)	Urban
Davies (2012)	Facilitating collaboration between primary care and public health	Analysis/examples of collaboration	Various nurse roles outlined	PH/PC leaders	Thesis/interviews	Canada (BC)	Both
Lebrun et al. (2012)	Primary care and public health activities in select U.S. health centers: documenting successes, barriers, and lessons learned	Health centre collaboration	Case management in public health role	PC/PH general	Mixed	USA	Both
Levesque et al. (2013)	The interaction of public health and primary care: functional roles and organizational models that bridge individual and population perspectives	Roles and models that support collaboration	Nurse role in PC teams	PC/PH general	Scoping Review	Canada (Quebec)	None
Peckham et al. (2011)	Health promotion and ill‐health prevention: the role of general practice	Understand how the two sectors interact	PC RNs role in health prevention and promotion	PC/PH general	Commentary	UK	None
Serpas et al. (2013)	San Diego Healthy Weight Collaborative: a systems approach to address childhood obesity	Chronic disease/collaboration Multisystem	System‐level RN works in PH collaborative	Multi‐agency	Commentary	USA	Urban
Levy et al. (2011)	The primary care nutrition training program: an approach to communication on behavior change	Collaborative project from PH to PC	System‐level RN role/programme facilitation	GP, MP, RN, SW, dieticians, MOA, clerical	Pre/Post‐test	USA	Urban
van Avendonk et al. (2012)	Primary care and public health a natural alliance? The introduction of the guidelines for obesity and undernutrition of the Dutch college of general practitioners	Practice guidelines	RN role screening for chronic disease in home setting	GP Practice Nurses Public Health nurses	Commentary	The Netherlands	None
Weinstein et al. (2013)	A primary pare‐public health partnership addressing homelessness, serious mental illness, and health disparities	Community partnership	Care coordination role in vulnerable population	PH, PC	Programme evaluation	USA	Urban
Kardakis et al. (2014)	Lifestyle interventions in primary health care: professional and organizational challenges	Health promotion in PC	PH versus PC nurses in PC had improved work in health promotion	GP, residents, PHNs and RNs in PC	Survey	Sweden	Rural

The number of papers from each country, Canada (*N* = 5), the USA (*N* = 9), Europe (UK, Ireland, Norway, Sweden, the Netherlands) (*N* = 6) and Australia (*N* = 3), was included.

PC/PH collaboration was evident at various stages from full collaboration to no collaboration. Studies from the USA (Bodenheimer, Chen, & Bennett, 2009; Elliott et al., 2014; Ferrer et al., 2013; Lebrun et al., 2012; Levy et al., 2011; Monsen et al., 2015; Serpas et al., 2013; Weinstein et al., 2013) and Canada (Davies, 2012; Green et al., 2013; Kates et al., 2012; Levesque et al., 2013; Wynn & Moore, 2012) report the youngest collaborative PC/PH systems versus studies from the European countries (van Avendonk, Mensink, Ton Drenthen, & van binsbergen, 2012; Clancy, Gressnes, & Svensson, 2013; Kardakis, Weinehall, Jerdén, Nyström, & Johansson, 2014; Kelly, Glitenane, & Dowling, 2015; Korhonen, Järvenpää, & Kautiainen, 2014; Peckham, Econ, Hann, & Hons, 2011), which discussed PC and PH systems that merged in the early 2000s.

The most common health areas of PC/PH collaboration that involved the RN’s role were maternal/child health and primary and secondary chronic disease prevention (Table 2). Many of the collaborations involved a targeted approach to vulnerable populations for health issues such as childhood obesity (Ferrer et al., 2013), access to maternity care for Indigenous women and other vulnerable populations (Davies, 2012; Psaila, Kruske, Kruske, Fowler, Homer, & Schmied, 2014), and chronic and communicable disease screening and prevention (Ferrer et al., 2013; Weinstein et al., 2013).

**TABLE 2:** Areas of focus for RN collaboration in primary care and public health

Health Focus	Reference
Chronic disease	Bodenheimer et al. (2009), Elliott et al. (2014), Ferrer et al. (2013), Green et al. (2013), Kates et al. (2012), Weinstein et al. (2013)
Maternal/child health	Clancy et al. (2013), Green et al. (2013), Psaila, Fowler, et al. (2014), Psaila, Fowler, et al. (2014), Psaila, Fowler, et al. (2014), Davies (2012)
Immunization	Kempe et al. (2014), Davis (2012), Levesque et al. (2013), Wynn and Moore (2012)
Obesity	Ferrer et al. (2013), Korhonen et al. (2014), Monsen et al. (2015), Serpas et al. (2013), Levy et al. (2011), van Avendonk et al. (2012)
Underserved populations	Davies (2012), Levesque et al. (2013), Weinstein et al. (2013)

No articles focused entirely on the role of the RN in PC/PH collaborations, and when this role was discussed, it was mentioned briefly. Four main RN roles were identified from the data: (a) relationship builder, (b) care coordinator, (c) outreach professional and (d) programme facilitator. These roles were identified at a variety of levels (systemic, organizational and interpersonal/intrapersonal), using the Ecological Framework (Appendix S2). For clarity, most of the RN roles discussed in the studies were community‐based public health nurses (PH RNs). The European studies’ primary care systems employed both primary care and public health nurses.

### 6.1 Relationship builder

At the intrapersonal/interpersonal level, PH RNs played an integral role in team‐based primary care and collaboration between PC and PH. Their role as frontline nurses, working with other providers, placed them in key roles to enhance communication through face‐to‐face interactions (Green et al., 2013; Kelly et al., 2015; Kempe et al., 2014; Monsen et al., 2015; Psaila, Kruske, et al., 2014; Wynn & Moore, 2012). Statements from PC providers about the PH RNs included words such as supportive, sharing, checking in, reminding, visiting and talking (Monsen et al., 2015). For example, a PC provider stated, “it gives us a face and a name so we can call (the public health department) if we have other problems” (Kempe et al., 2014, p. 115). From the PH perspective, it was stated “I think the better they (PC providers) know us and the more they see us as an actual resource, the more comfortable they are when there's really a public health issue that has to be dealt with” (Kempe et al., 2014, p. 115).

At the organizational level, PH RNs built relationships with organizations in and outside the healthcare sector to improve access to care by providing PC in locations where people work, live and play (Kempe et al., 2014) rather than at stationary PC sites. As PH RNs spent time and resources to support PC staff in their practice setting, face‐to‐face communication between PH and PC was enhanced and described as a “network of communication” by Wynn and Moore (2012), where future initiatives between PC and PH such as chronic disease management and disease surveillance could be supported.

### 6.2 Care coordinator

At the intra/interpersonal level, the care coordinator role was seen in collaborative clinics and hospital community transition programmes. It was often motivated by the need to increase access to care for vulnerable individuals and families, particularly in maternal/child and chronic disease management health services (Davies, 2012; Ferrer et al., 2013; Psaila, Fowler, Kruske, & Schmied, 2014). Evidence from these studies suggests that PH and PC RN care coordinators improved access to care, streamlined services and increased referrals to community services. RNs integrated care between hospital and community, ran group visits to support chronic disease management and maternal care and addressed the social determinants of health (Ferrer et al., 2013).

At the organizational level, care coordination occurred with PH RNs collaborating with community agencies (Elliott et al., 2014) and working with PC to improve vertical and horizontal continuity of care, specifically in addressing the social determinants of health for complex maternity patients (Davies, 2012; Psaila, Fowler, et al., 2014) and chronic disease management (Ferrer et al., 2013; Weinstein et al., 2013).

### 6.3 Outreach professional

At the intra/interpersonal level, the literature showed that the PH RN’s role as an “outreach professional” improved patient access to PC, improved prevention and supported a variety of community service and care models. This role is particularly relevant to vulnerable populations. For example, outreach to home settings (only seen in European studies) targeted primary and secondary prevention of chronic disease and obesity. RN activities included assessment and education (Korhonen et al., 2014), monitoring of food intake and body weight and implementation of advice from a dietician (van Avendonk et al.., 2012). RN‐led screening achieved meaningful weight loss with brief lifestyle counselling (Korhonen et al., 2014).

At the organizational level, this outreach care was often associated with team‐based care models such as the Primary Care Medical Home. This care delivery model provides comprehensive and coordinated patient care by providers such as physicians, nurses, pharmacists and social workers (www.pcmh.ahrq.gov). Public health RNs worked either as consultants or as members of Primary Care Medical Home models and often in association with academic collaborative PC settings (Weinstein et al., 2013). An example of this approach occurred during the H1N1 pandemic in 2010 where family health teams (PC) and PH RNs collaborated in the management of a flu pandemic by creating assessment centres in the community to ensure universal and easy access to care. At these sites, PH RNs liaised with PC sites to provide supplemental staffing for flu immunization clinics and infection control measures such as cough etiquette, education and quarantine, and coordination of clinical care guidelines (Wynn & Moore, 2012).

Beyond traditional healthcare sites, PH RN‐led outreach immunization clinics improved access to care and increased vaccination rates by providing immunizations at community‐based venues allowing care to be provided in unique locations and unusual times (Kempe et al., 2014; Lebrun et al., 2012). Notably, the largest immunization rate increase was seen among healthy children who do not regularly interface with the PC system. This highlights a unique feature of the RN’s role in communicable disease reduction by immunization outreach to people who face barriers in access or do not regularly access PC (Kempe et al., 2014).

### 6.4 Programme facilitator

At the organizational level, the PH RN programme facilitator role supported the transfer of PH knowledge to PC in areas such as communicable disease (immunization and pandemic management) (Green et al., 2013; Kempe et al., 2014; Wynn & Moore, 2012) and chronic disease prevention, for example healthy eating strategies (van Avendonk et al., 2012; Levy et al., 2011; Monsen et al., 2015). The RN’s role at this level not only supported interorganizational collaboration, but also enabled future collaboration and increased awareness of population‐level needs in PC (Kempe et al., 2014; Levy et al., 2011; Monsen et al., 2015; Wynn & Moore, 2012).

At the system level, the RN’s role supported the use of tools, guides and programmes in PC/PH collaboration. Examples of RNs working at the system level include the following: (a) supporting the use of the Institute for Systems Improvement—Adult Obesity Guideline into the PC setting in Minnesota (Monsen et al., 2015); (b) implementing the Primary Care Nutrition Training Program to targeted underserved PC sites in New York (Levy et al., 2011); (c) liaising with schools and community organizations and PC to implement weight screening and healthy eating with The San Diego Healthy Weight Collaborative (Serpas et al., 2013); and (d) assisting PC obesity guideline translation, including goal setting with PC sites in the use of guidelines and tools, educating PC site staff on motivational interviewing techniques, supplying resources (food models and portion control plates) and facilitating shared best practices in obesity management (Monsen et al., 2015).

### 6.5 RN scope of practice supported RN’s role identified in the scoping review

The roles identified in this scoping review are in legal regulations in the RN scope of practice (British Columbia College of Nursing Professionals (BCCNP) 2018). However, as new roles emerge in the face of changing healthcare demands, challenges with scope of practice documents maintaining their currency have been noted internationally (Birks, Davis, Smithson, & Cant, 2016; Fealy et al., 2015).

*Conclusion*. In supporting new roles, regulatory bodies can work with nurse associations and health authorities to advance the concept of individual‐level accountability and self‐reliance in determining best practice decisions.

These results suggest a growing body of evidence of RNs working to strengthen collaboration across PC and PH sectors. We provide evidence to extend our understanding around the various roles RNs can undertake and the kinds of activities they can perform in their scope of practice. Most of what RNs are undertaking in strengthening collaboration across PC and PH sectors occurs at the inter/intrapersonal and organizational levels. Worldwide, RNs play an ever important role in delivering primary healthcare services (Bauer & Bodenheimer, 2017; Smolowitz et al., 2015), and given the rise of health and social inequities globally (OECD, 2017; WHO, 2008), these roles and activities strengthen the primary healthcare system by increasing health promotion and prevention and access to care (OECD, 2017).

RNs contributed to improved organizational‐level collaboration through increased personal connections and information communication pathways (Levy et al., 2011; Psaila, Kruske, et al., 2014; Wynn & Moore, 2012). Past work suggests that building and sustaining relationships can be more important than structural considerations such as colocation (Clancy et al., 2013; Kempe et al., 2014). Termed “boundary spanners” by Fraher et al. (2015), RNs can perform new roles for a new healthcare system with a population health focus. Improved care coordination and transition between the sectors can be supported by these RN roles, highlighting their importance in change management process that occurs with any healthcare reform efforts. As outreach professionals and care coordinators, RNs increased access to PC and PH especially with populations made vulnerable by multiple intersecting determinants of health such as those living on or close to the street and facing homelessness. This role is key to future in the management of disease outbreaks. Programme facilitator roles were particularly important at the organizational and systemic levels. This scoping review reveals the importance of the primary healthcare RN to be used beyond the walls of the office setting to provide care in the community from interpersonal to interorganizational and system levels.

We found less evidence of RN roles in PC and PH at the system level. There are many political, policy, structural and workforce barriers that prevent the expansion of the RN role to collaborative work at the interorganizational and system levels. Societal attitudes, government policy and structural barriers that include the current GP private practice and lack of funding models to support PC nursing (APNA, 2017) and perceived fiscal constraints all constrain RN roles. In PC transformation, RN role development will be under pressure from these complex factors. Without due consideration in role development, as has occurred in Australia and Ireland (Brookes, Daly, Davidson, & Halcomb, 2007; Kelly et al., 2015), RN roles may devolve into roles and activities that focus on “acute care” delivered in the community missing out on important health prevention and promotion activities.

In performing collaborative roles, RNs were more commonly employed by public health than RNs working in PC practice. This could be due to their specific PH RN expertise such as collaboration, community interface/outreach and relationship building and the small number of generalist PC RNs in North American PC settings (Martin‐Misener & Bryant‐Lukosius, 2014; Valaitis et al., 2013). Structural issues such as the fee‐for‐service remuneration model and the fact that the PC system is separate from the hospital/PH system also act as barriers to employing more RNs in primary care (Bauer & Bodenheimer, 2017). Further research to support the RN roles includes evaluation of the RN’s basic education preparation specific to these roles.

To ensure that nurses are able to work in these roles, awareness of the RN scope of practice by government and health authorities and PC providers is essential, and as Bauer and Bodenheimer (2017) suggest, reversing overly cautious interpretations of scope of practice regulations. Inclusion of primary healthcare content and clinical practice into pre‐licensure training will ensure RNs are well prepared for skills in cross‐sector collaboration and primary care (Bodenheimer & Mason, 2017). Ensuring that nursing educators have PC experience could support the establishment of theory and practice into these unique roles and skills into nursing education (Fraher et al., 2015). Opening opportunities for RNs in new models of primary care (Hutchison et al., 2011) and improving RNs in supporting their own role development will also support appropriate nurse's roles in PC (Ashley, Halcomb, Brown, & Peters, 2018).

## 7 CONCLUSION

There are limitations to this scoping review that require consideration. There was an opportunity of some literature being missed due to database selection, search limitations in language and country and possibly missing some grey literature. There was also a lack of depth in the broadscale nature of the scoping review, and no articles specifically about nurse's roles were obtained, which may have affected the results of the study. The focus of scoping reviews is to provide breadth rather than depth of information; thus, a meta‐analysis is generally not conducted (Tricco et al., 2016). However, this method was appropriate for our research given our objectives and the current broad understanding of this topic. Finally, it is possible that there are additional roles for RNs in PC/PH collaboration, as our review included work up to 2016. However, the RN roles described here form a foundation for any new and emerging roles.

Strengthening PC/PH collaboration supports a population health approach and targeted prevention strategies called for by multiple agencies throughout the world (OECD, 2017; WHO, 2017). RN roles in PC/PH collaboration, in terms of both the activities performed and populations served, could strengthen cross‐sector work and therefore increase the effectiveness of the primary healthcare system in addressing the healthcare needs of the population. This collaborative role is particularly relevant to vulnerable populations whose health and social needs are optimally met through a team‐based, patient‐centred approach (Browne et al., 2012). RN’s roles and activities in PC/PH can support greater continuity of care and health promotion through individual‐, organizational‐ and systemic‐level interventions.

RNs act as the “glue” between PC and PH for patients, providers, organizations and health systems. This role is important in reducing gaps in care and improving health outcomes, especially for vulnerable populations. To optimize the RN role in PC and PH collaboration, government and health authorities should enhance their understanding of the competencies and scope of practice of RNs (APNA, 2017; Canadian Nurses Association, 2015; Fraher et al., 2015; Halcomb et al., 2016) and resources should be provided to support professional development for RNs in new and emerging roles. When designing effective primary care teams, policymakers should consider these diverse and varied RN roles and activities and ensure that they are allowed to flourish in a flexible and responsive primary healthcare system with improved health outcomes.

## CONFLICT OF INTEREST

The authors have no conflict of interest to declare.

## Supporting information

Appendix S1‐S2

Suppinfo
