# Supplementary material for: The role of registered nurses in primary care and public health collaboration: A scoping review
Source: Nurs Open. 2020 Apr 14;7(4):1197–207. doi: 10.1002/nop2.496 (PMC7308712; doi:10.1002/nop2.496)
Supplement: Supplementary file 1 — Appendix S1‐S2 [file NOP2-7-1197-s001.docx]

**Appendix A**

**Initial Search Strategy**

The UBC Health Sciences librarian was consulted to develop the initial search strategy (Tables 1-3). Dr. Wong was also consulted to verify search strategy. Testing of the strategy yielded appropriate literature.

**Dates:** Peer reviewed articles and grey literature from May 2008 to Jan. 2016.

**Geographic inclusion**: United States, Canada, Western Europe, Australia and New Zealand.

**Inclusion/Exclusion criteria**:

Address at least one of the following: collaborative teams PC/PH, nurse’s role in PC/PH. Papers will be excluded if they only address PH or PC alone and did not address nurse’s role and were not published in English.

*Table 1 – Search Strategy*

| **Search strategy** |
| --- |
| Electronic Databases: PubMed, CINAHL, Cochrane, PsycInfo, and Dissertation International; |
| Searching reference lists of key documents; |
| Web search of government, association and research networks will be scanned for key documents and information; |
| General internet search using GOOGLE; |
| Hand search of relevant journals; and |
| Liaising with key informants. |

MeSH headings were used as free text key words: PC, PH, collaboration, public health nurse, community health nurse, nurse, patient care teams, and nurse’s roles using ‘AND’ and ‘OR’. Search terms were established from target literature that was a good representation of the research question. Subject headings applied to the article were noted and mesh headings were established for the search strategy. Using the same subject headings, additional articles were found.

*Table 2 - Search terms:*

| **Professional name** | **Organization** | **Structure** | **Function** | **Method** |
| --- | --- | --- | --- | --- |
| Community Health Nurse  Public Health Nurse  Nurse | Primary Care  Primary Health Care  Public Health | Health Care Teams  Patient Care Teams | Nurse’s Role | Collaboration |

*Table 3 - Mesh heading confirmation:*

| Concept | Title | Key words Mesh Synonyms | **Mesh Definition** |
| --- | --- | --- | --- |
| Nurse | Community Health Nurse | Community Health NurseHome care nurseHome visitorHome Health Nurse | Nurses whose work combines elements of both primary care nursing and public health practice and takes place primarily outside the therapeutic institution. Primary nursing care is directed to individuals, families, or groups in their natural settings within communities. |
|  | Public health nurse | (no synonyms) | Nurses whose goal is to improve health and quality of life in a population or community through the prevention and treatment of disease and other physical and mental health conditions, the surveillance of cases and health indicators, and the promotion of healthy behaviours through public education and awareness. |
|  | Nurse | Nurse  Nursing personnel  Registered nurse | Professionals qualified by graduation from an accredited school of nursing and by passage of a national licensing examination to practice nursing. They provide services to patients requiring assistance in recovering or maintaining their physical or mental health. |
| **Health system component** | Primary Health Care | Primary Care  Primary Health Care  Primary healthcare | Care which provides integrated, accessible health care services by clinicians who are accountable for addressing a large majority of personal health care needs, developing a sustained partnership with patients, and practicing in the context of family and community. |
|  | Public Health | Public Health | Branch of medicine concerned with the prevention and control of disease and disability, and the promotion of physical and mental health of the population on the international, national, state, or municipal level. |
| **Organization** | Patient care teams | Patient care teams  Health care teams Interdisciplinary teams | Care of patients by a multidisciplinary team usually organized under the leadership of a physician; each member of the team has specific responsibilities and the whole team contributes to the care of the patient. |
| **Method** | Collaboration | Cooperative teams  Collaboration | The interaction of two or more persons or organizations directed toward a common goal, which is mutually beneficial. An act or instance of working or acting together for a common purpose or benefit, i.e., joint action. |
| **Nurse’s Role** | Nurse’s role | No synonyms | The expected function of a member of the nursing profession. |

**Appendix B**

Primary Care/Public Health Frameworks/Models of Collaboration


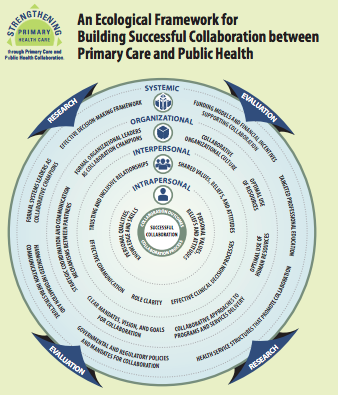
The Ecological Framework for Building Successful Collaboration Between Primary Care and Public Health (Valaitis et al., 2013, p.44).
